# The latent structure of cognitive and emotional empathy in individuals with autism, first-degree relatives and typical individuals

**DOI:** 10.1186/2040-2392-5-42

**Published:** 2014-08-01

**Authors:** Rachel Grove, Andrew Baillie, Carrie Allison, Simon Baron-Cohen, Rosa A Hoekstra

**Affiliations:** 1 Centre for Emotional Health, Department of Psychology, Macquarie University, 2109 Sydney, NSW, Australia; 2 Autism Research Centre, Department of Psychiatry, Cambridge University, Cambridge, UK; 3 CLASS Clinic, Cambridgeshire and Peterborough NHS Foundation Trust, Cambridge, UK; 4 Faculty of Science, The Open University, Milton Keynes, UK

**Keywords:** Empathy, autism, broader autism phenotype, factor analysis

## Abstract

**Background:**

Empathy is a vital component for social understanding involving the ability to recognise emotion (cognitive empathy) and provide an appropriate affective response (emotional empathy). Autism spectrum conditions have been described as disorders of empathy. First-degree relatives may show some mild traits of the autism spectrum, the broader autism phenotype (BAP). Whether both cognitive and emotional empathy, rather than cognitive empathy alone, are impaired in autism and the BAP is still under debate. Moreover the association between various aspects of empathy is unclear. This study aims to examine the relationship between different components of empathy across individuals with varying levels of genetic vulnerability to autism.

**Methods:**

Factor analyses utilising questionnaire and performance-based task data were implemented among individuals with autism, parents of a child with autism and controls. The relationship between performance-based tasks and behavioural measures of empathy was also explored.

**Results:**

A four-factor model including cognitive empathy, emotional empathy, social skills and a performance-based factor fitted the data best irrespective of genetic vulnerability. Individuals with autism displayed impairment on all four factors, with parents showing intermediate difficulties. Performance-based measures of empathy were related in almost equal magnitude to cognitive and emotional empathy latent factors and the social skills factor.

**Conclusions:**

This study suggests individuals with autism have difficulties with multiple facets of empathy, while parents show intermediate impairments, providing evidence for a quantitative BAP. Impaired scores on performance-based measures of empathy, often thought to be pure measures of cognitive empathy, were also related to much wider empathy difficulties than impairments in cognitive empathy alone.

## Background

Empathy has been defined as the drive to identify and respond appropriately to emotions and mental states in others [1,2]. It plays a vital role in human relationships and allows an individual to make sense of and predict the behaviour of another [3]. Empathy involves both the ability to recognise and understand emotion in others [3] as well as an affective response to another’s emotional state [4,5], respectively cognitive and emotional empathy [4,6].

Autism spectrum conditions (ASC) involve empathy deficits [6-9] and are characterised by communication and social difficulties as well as repetitive behaviours or restricted interests [10]. Empathy dysfunction in autism has been demonstrated via research noting a theory of mind (ToM) impairment in children with ASC [11]; that is, that individuals with autism have difficulty reading the beliefs and intentions of others [11,12]. ToM is often used interchangeably with cognitive empathy, perspective taking and ‘mentalising’ [13]. However, as noted above, empathy has long been defined as a multifactorial construct including not only the representation of another’s emotional state (ToM or cognitive empathy) but also an affective response (emotional empathy).

The Empathising-Systemising (E-S) theory [14,15] expands the concept of ToM to include this affective component of empathy. The E-S theory argues that the social and communication difficulties seen in ASC can be accounted for by an empathy impairment (including both cognitive and emotional components) and the repetitive behaviours and narrow interests by an inclination for systemising (the drive to understand and derive rules about a system) [16]. A recent factor analytic study by the authors [17] found support for the E-S model. This study, based on the same individuals as the research reported here, identified two factors representing empathy and systemising. These factors were found consistently across individuals with autism, first-degree relatives and general population controls [17]. In concordance with the E-S theory, individuals with ASC showed elevated scores on the latent systemising factor and low scores on the empathy factor. This previous study included questionnaire measures of empathy and systemising only. However, other studies have indicated that individuals with autism also have difficulty with performance-based tasks involving the identification of emotions and perspective taking [18-20]. As these tasks involve the identification of emotion, they are generally conceptualised as performance-based tasks of cognitive empathy.

Although there is much evidence to suggest that individuals with autism display difficulties with ToM or cognitive empathy, there is more debate about the role of emotional empathy in autism. While mirror neuron theory [21] argues that individuals with ASC have weak emotional empathy, Dziobeck and others [22] claim that emotional empathy is intact in autism. Other theorists have proposed that it is due to heightened emotional empathy that individuals with ASC find the social world more challenging, arguing that it is overwhelming rather than difficult to understand [3,23,24].

First-degree relatives of individuals with an ASC diagnosis may also show some mild traits of the autism spectrum [25], also referred to as the broader autism phenotype (BAP) [26,27]. The finding of the BAP fits with the notion that autism is under polygenic influence, and that at least part of these genetic influences are inherited (rather than *de novo* genetic events) and can also be found in undiagnosed relatives displaying the broader phenotype [28]. The BAP has also been shown to apply to empathy, with parents and siblings of affected individuals scoring lower on performance-based tasks involving emotion recognition [20,29-31] and questionnaire measures assessing empathy [20,27]. It is therefore important to examine cognitive and emotional empathy not only in clinical samples, but across the full range of genetic variability, including individuals on the autism spectrum, their relatives and general population controls.

A number of quantitative measures of empathy have been used in previous research, including the Interpersonal Reactivity Index [32] and the Empathy Scale [33]. However, one of the most widely used measures is the Empathy Quotient (EQ) [6], a self-report measure of empathy assessing both cognitive and emotional components. The EQ has recently been studied in detail across three studies. Two studies highlight a three-dimensional structure including cognitive empathy, emotional empathy and social skills [34,35], with the third highlighting a single dimension [36]. The first two studies were based on student and general population samples, with the third including individuals with autism and first-degree relatives. Although individuals with ASC and family members were included in the third study, the factor structure and utility of the EQ was examined for the whole sample and not for each of the three groups (individuals on the spectrum, first-degree relatives and general population controls) separately.

Although the EQ and performance based measures of cognitive empathy have been studied quite extensively by themselves in previous studies, the relationship between subscales of the EQ (a questionnaire-based measure), and performance-based measures of empathy have not been comprehensively assessed to date. The current study aims to evaluate the multifactorial nature of empathy utilising both behavioural and performance-based task data. It was assessed whether the latent structure of empathy differs across samples stratified by genetic vulnerability (individuals with ASC, first degree relatives and controls).

## Methods

### Participants

Individuals were recruited via two online databases from the Autism Research Centre (http://www.autismresearchcentre.com) and the Department of Psychology (http://www.cambridgepsychology.com) at the University of Cambridge. The total sample consisted of 1,034 community-based participants including individuals with ASC (193 males, 170 females; mean age, 36 years; sd, 11), parents of a child with ASC (141 males, 298 females; mean age, 42 years; sd, 8) and general population controls (122 males, 110 females; mean age, 33 years; sd, 10). Individuals who reported no previous psychiatric history were included in the control group. Individuals who had a formal ASC diagnosis were included in the autism group. The control group contained a significantly larger proportion of individuals with an undergraduate degree than the parent and ASC groups (*P* <0.001). Ethics approval for data collection was given by the Cambridge Psychology Research Ethics Committee and all participants gave informed consent prior to taking part in the study.

### Measures

#### Empathy

The EQ [6] is a self-report measure assessing both cognitive (for example, ‘I can tune into how someone else feels rapidly and intuitively’) and emotional empathy (for example, ‘seeing people cry does not really upset me’). The EQ includes 40 statements with four response options; ‘strongly disagree’, ‘slightly disagree’, ‘strongly agree’, and ‘slightly agree’. ‘Strongly agree’ responses are given 2 points, with ‘slightly agree’ responses receiving 1 point. Higher scores are indicative of increased levels of self-reported empathy. The EQ shows good test-retest reliability (*r* = 0.97, *P* <0.001) [6].

#### Autistic traits

The Autism Spectrum Quotient (AQ) [37] assesses quantitative autistic traits including communication, imagination, attention to detail, social skills and attention switching. Fifty items are assessed on a 4-point Likert scale with response categories ‘definitely disagree’, ‘slightly disagree’, ‘definitely agree’ and ‘slightly agree’. Hoekstra and others [38] outline a raw scoring method, with total scores in the range of 50 to 200; higher scores indicating the presence of autistic traits. Previous research has highlighted that the AQ shows good test-retest reliability [37].

A previous factor analysis showed that the AQ can be reliably split into two factors assessing social and non-social autistic traits [38]. A broad social interaction factor was compiled using items assessing communication, social skills, imagination and attention switching (40 items). As the focus of the current study is on empathy, the further 10 items assessing attention to detail or non-social autistic traits were excluded from the current analysis.

#### Performance tasks

The ‘Reading the Mind in the Eyes’ test revised [39] is a performance task designed to assess how well an individual can read another’s emotion based on viewing the eye area alone. This measure has been described as an advanced ‘theory of mind’ task that assesses the ability to attribute mental states to oneself and others (i.e. cognitive empathy). Individuals are presented with a series of 36 photographs of the eye region of the face and asked to choose which of four words best describes the emotion depicted. The emotions used in the task are subtle and include, for example, a choice between jealous, panicked, arrogant and hateful. This test has been shown to detect meaningful individual differences, with individuals with AS or HFA scoring significantly lower than general population controls [39].

The Karolinska Directed Emotional Faces (KDEF) [40] is another task designed to assess the recognition of more basic emotions in others. In this modified version, participants were shown 140 photographs of faces expressing seven emotions (happy, sad, angry, afraid, disappointed, surprised and neutral). For each photograph, individuals were asked to select which of the seven emotions best described the emotion depicted. Results provide indications of accuracy and response time for each facial expression. Accuracy adjusted response time was calculated by dividing the mean response time for correct items by the proportion of items answered correctly. Weighted mean reaction times have been shown to be a more sensitive measure, taking any potential speed-accuracy trade off into account [41]. The KDEF has good test-retest reliability and has been validated on emotional content, intensity and arousal [42]. Individuals with autism have been shown to score lower than controls on this task [20]. To aid data interpretation, the KDEF was rescored so that lower values indicate higher accuracy adjusted response time and hence lower empathy ability.

Given that sex differences on the mean test scores were not the focus of this paper and have been reported elsewhere [20,38,39], any effects of sex and age on the mean test scores were regressed out prior to factor analysis. This enabled the comparison of the factor structure of empathy without the confound of sex differences on the mean. Furthermore, the standardisation of the items allowed for any differences in variance between the items of the EQ and the Eyes and KDEF tasks to be accounted for (as standardisation resulted in all variables having a mean of 0 and a variance of 1).

### Analytic strategy

Previous research has shown that the EQ can be split into three factors: cognitive empathy, emotional empathy and social skills [34,35]. Although finding a comparable factor structure, these two papers showed differences in the number of items loading onto each latent factor. Allison et al. [36] have also explored the EQ in depth, highlighted a single dimension using Rasch analyses. The first stage of our analyses focused on determining the most appropriate factor structure for the EQ in the current sample.

Confirmatory factor analyses (CFA) were conducted in Mplus Version 7 using the maximum likelihood estimator [43]. Confirmatory models allow for a more direct test of previous models of empathy as well as greater control over model specification. The first set of analyses assessed the fit of a one-factor 26-item model (following the model identified by Allison et al. [36]) across: (1) individuals with autism; (2) parents; and (3) general population controls (Models 1 to 3). Following this, three-factor models assessing cognitive empathy, emotional empathy and social skills were estimated based on Lawrence et al.’s [34] 28-item model (Models 4 to 6) and Muncer et al.’s [35] 15-item model (Models 7 to 9). The best fitting model identified in each of the three groups separately was then subjected to multiple group analysis to determine whether the same latent structure holds across individuals with autism, parents and controls (Models 10 and 11). In all subsequent analyses, the model that best described the EQ data across all three groups, a three-factor model including factors assessing cognitive empathy, emotional empathy and social skills (see Results section) was utilised.

Following the analysis of the EQ alone, the study of the latent structure of empathy was extended by also including the AQ, Eyes and KDEF measures in the factor analysis. The AQ was not submitted to rigorous individual investigation as it has previously been studied extensively [37,38,44-48].

First, a series of three-factor models (with latent factors Cognitive empathy, Emotional empathy and Social skills) were tested. The social interaction factor of the AQ was predicted to load on the Social skills factor due to the similarity between the content of the AQ items and the EQ items loading on this factor. The Eyes and the KDEF scores were expected to load onto the Cognitive empathy factor of the EQ as these two performance tests are thought to measure cognitive empathy (Models 12 to 14). Second, a series of four-factor models were estimated, in which the Eyes and KDEF scores loaded on to a separate fourth measurement factor representing performance-based assessment of empathy, rather than on the Cognitive empathy factor (Models 15 to 17). Multiple group models were used to determine whether the same structure was present among individuals with autism, parents and general population controls. The first multigroup CFA allowed all parameters to vary across the three groups (Model 18). A further model constraining the factor loadings to be equal across groups was also tested (Model 19).

In order to evaluate the possible impact of sex differences on the latent structure of empathy, three further models incorporating six groups based on genetic vulnerability (ASC *vs.* parents *vs.* controls) and sex (males *vs.* females) were assessed using multigroup CFA (Models 20 to 22). As before, these models were run using test scores corrected for any mean sex (and age) differences, to ensure that these models focused on possible sex differences in latent structure, rather than sex differences in mean test scores. A number of fathers in the dataset had missing data on the performance-based tasks (n = 104). In order to account for the effect of this missing data on the results, all six-group analyses were run both by imputing the data for these individuals as well as excluding these individuals for comparison.

Model fit was evaluated using the Bayesian information criterion (BIC) [49], Sample size adjusted BIC (SSABIC) [50], Akaike information criterion (AIC) [51], Tucker-Lewis index (TLI) [52], Comparative fit index (CFI) [53] and the Root mean square error of approximation (RMSEA) [54]. The BIC, SSABIC and AIC are used to assess model fit, with lower values reflective of a more parsimonious model. TLI and CFI compare the model under investigation with the null model, with CFI and TLI values > = 0.95 indicating very good fit and values > = 0.90 representing adequate fit [55,56]. The RMSEA is a fit index that allows for modelling with large sample sizes. RMSEA values <0.08 indicate adequate fit, with values <0.05 suggesting excellent fit [57]. Evaluation of model fit also included the interpretability of all other parameter estimates. Comparison of the nested models was based on chi-square difference tests. These have been shown to result in less type one error when the maximum likelihood estimator is implemented [58].

## Results

### Factor analyses of empathy as assessed by items of the EQ

Model fit indices ascertained from the CFA models are given in Table 1. The model describing a one-factor solution of the EQ data, following Alison et al.’s [36] model, displayed poor fit in all three groups (Models 1 to 3 in Table 1). Similarly, fit indices based on Lawrence et al.’s [34] three-factor model of the EQ were below recommended thresholds (Models 4 to 6). The three-factor model of the EQ based on Muncer et al. [35] provided the best fit to the data (Models 7 to 9). Multigroup CFA analyses indicated that this model displayed good fit across individuals with autism, parents and general population controls (Model 10). A model in which the factor loadings were constrained to be equal across the three groups (Model 11) resulted in a significantly poorer fit compared with Model 10 (*χ*^2^ = 114.1, *P* <0.001). These findings suggest that the EQ assesses three constructs (Cognitive empathy, Emotional empathy and Social skills) in controls, parents and adults on the autism spectrum, but the association between each of these latent constructs is somewhat different across the three groups. For example, the factor correlations in the ASC group were higher than controls. This may account for why Model 11 did not provide a good fit to the data.

**Table 1:** Fit indices and model comparisons

**Model**	**Description**	**Fit indices**							
		**AIC**	**BIC**	**SSABIC**	**RMSEA**	**CFI**	**TLI**	***χ***^**2**^	**Δχ**^**2 **^**( *df *)**
**One-factor models EQ items (Allison et al., 2011 **[36]**)**									
1	1f control group (n = 232)	13,673.951	13,942.797	13,695.578	0.090	0.673	0.644	865.2^a^	
2	1f parent group (n = 439)	25,650.600	25,969.013	25,721.480	0.083	0.833	0.818	1,193.9^a^	
3	1f autism group (n = 363)	22,451.458	22,755.222	22,507.762	0.087	0.748	0.726	1,123.5^a^	
**Three-factor models EQ items (Lawrence et al., 2004 **[34]**)**									
4	3f control group (n = 232)	14,020.059	14,319.925	14,044.181	0.078	0.781	0.761	833.2^a^	
5	3f parent group (n = 439)	26,186.672	26,541.825	26,265.731	0.071	0.883	0.873	1,114.3^a^	
6	3f autism group (n = 363)	23,768.627	24,107.440	23,831.427	0.080	0.802	0.785	1,163.0^a^	
**Three-factor models EQ items (Muncer et al., 2006 **[35]**)**									
7	3f control group (n = 232)	7,731.634	7,897.077	7,744.943	0.048	0.942	0.930	132.7^a^	
8	3f parent group (n = 439)	14,798.686	14,994.632	14,842.304	0.060	0.950	0.939	222.0^a^	
9	3f autism group (n = 363)	13,040.850	13,227.781	13,075.499	0.055	0.932	0.918	184.3^a^	
**Three-factor multigroup models EQ items (Muncer et al., 2006 **[35]**)**									
10	3f multigroup all estimates vary (n = 1,034)	35,574.089	36,166.916	35,785.782	0.056	0.938	0.931	589.9^a^	
11	3f multigroup equal factor loadings (n = 1,034)	35,628.149	36,072.769	35,786.918	0.060	0.921	0.921	703.9^a^	114.1 (30) *P* <0.001
**Three-factor model of cognitive empathy (EQ subscale, Eyes and KDEF), emotional empathy (EQ subscale) and social skills (EQ subscale and AQ_soc)**									
12	3f control group (n = 232)	8,331.507	8,527.971	8,347.312	0.044	0.945	0.936	1,202.4^a^	
13	3f parent group (n = 439)	16,721.291	16,954.108	16,773.218	0.055	0.947	0.938	3,463.6^a^	
14	3f autism group (n = 363)	19,949.121	20,171.102	19,990.266	0.067	0.894	0.877	2,162.7^a^	
**Four-factor model of cognitive empathy (EQ subscale), emotional empathy (EQ subscale) social skills (EQ subscale and AQ_soc) and performance-based empathy (Eyes and KDEF)**									
15	4f control group (n = 232)	8,323.897	8,530.701	8,340.533	0.040	0.955	0.946	176.4^a^	
16	4f parent group (n = 439)	16,712.141	16,957.074	16,766.664	0.055	0.949	0.939	298.8^a^	
17	4f autism group (n = 363)	15,687.606	15,921.270	15,730.916	0.053	0.934	0.922	262.5^a^	
**Four-factor multigroup models of cognitive empathy (EQ subscale), emotional empathy (EQ subscale) social skills (EQ subscale and AQ_soc) and performance-based empathy (Eyes and KDEF)**									
18	4f multigroup all estimates vary (n = 1,034)	40,743.301	41,494.361	41,011.591	0.052	0.938	0.932	808.0^a^	
19	4f multigroup equal factor loadings (n = 1,034)	40,894.787	41,467.965	41,099.535	0.061	0.909	0.907	1,031.5^a^	223.5 (36) *P* <0.001
**Four factor multigroup models specifying sex effects (6 groups)**									
20	4f multigroup all estimates vary (n = 1,034)	40,757.204	42,190.149	41,269.074	0.058	0.923	0.916	1,332.5^a^	
21	4f multigroup equal factor loadings and equal variance estimates (n = 1,034)	40,873.332	41,861.570	41,226.345	0.066	0.891	0.893	1,628.6^a^	296.1 (90) *P* <0.001
22	4f multigroup equal factor loadings and free variance (n = 1,034)	40,745.441	41,832.503	41,133.757	0.059	0.914	0.914	1,460.7^a^	128.2 (70) *P* <0.001

^a^*P* <0.001.

AIC, Akaike information criteria; AQ_soc, social interaction factor of the Autism Quotient; BIC, Bayesian information criteria; CFI, Comparative fit index; Eyes, Items correct on Reading the Mind in the Eyes Task; KDEF, Weighted mean reaction time on the Karolinska Directed Emotional Faces task; RMSEA, Root mean square error of approximation; SSABIC, Sample size adjusted BIC; TLI, Tucker-Lewis index; *χ*^2^, chi square statistic; Δχ^2^ (*df*), chi square difference test.

### Factor analyses including both behavioural and performance-based measures of empathy

Next, the Eyes and KDEF tasks and the social interaction factor of the AQ were included in Muncer et al.’s [35] three-factor model of empathy. First, it was tested whether the performance-based tasks solely assess cognitive empathy, by including these two variables in the Cognitive empathy factor (Models 12 to 14). In these models, factor loadings of the Eyes and KDEF on the cognitive empathy factor were not statistically significant. This poor fit was also reflected in some of the fit indices, with CFI and TLI values under the recommended threshold in the ASC group. Second, a model in which the KDEF and Eyes data loaded onto a separate ‘performance-based test factor’ was implemented. This four-factor model (including factors Cognitive empathy, Emotional empathy, Social skills and a Performance-based test factor) provided a good fit to the data in all individual groups (Models 15 to 16) as well as within the multigroup analysis (Model 18). Again, the model in which the factor loadings were constrained to be equal across the three groups (Model 19) resulted in a significantly poorer fit compared to the freely estimated model (Model 18) (*χ*^2^ = 223.5, *P* <0.001).

### Assessing sex differences in the factor structure of empathy

Lastly, the impact of sex on the factor structure of empathy was explored by running six-group analyses for the best-fitting model identified, the four-factor model including factors Cognitive empathy, Emotional empathy, Social skills and a Performance-based test factor. As the effects on the mean test scores were regressed out prior to analysis, these models (Models 20 to 22) focus on sex differences in factor structure rather than in mean scores. In order to account for the effect of missing data on the performance-based tasks, all six-group analyses were also run both by imputing data for and excluding these individuals. There were no substantive changes in any of the analyses, indicating that this is not a confounding factor in the interpretation of the results. Model 20 with all estimates free to vary provided the best fit to the data for the six groups. However, comparison with the best fitting three-group multigroup model (Model 18) indicates that there is no significant difference between the latent structure of empathy when sex is taken into account. Therefore, Model 18, the four-factor model with equal form among the three groups, allowing the factor loadings to vary provided the best fit to the data.

### Empathy factor means and correlations in individuals with ASC, parents and controls

**Figure 1:**
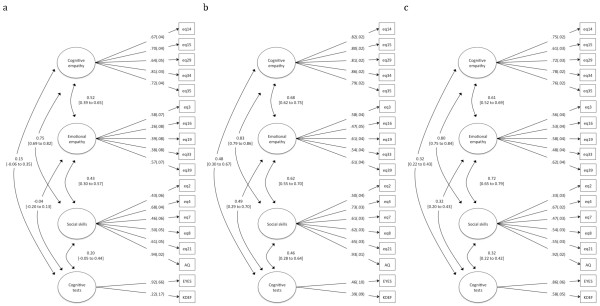
Four-factor multigroup model for (a) controls, (b) parents and (c) ASC.

Parameter estimates for the four-factor model taken from the Model 18 analysis are given in Figure 1. All items loaded significantly onto their respective factors (*P* <0.05). Mean differences between scores on the latent factors across the three groups are given in Table 2. Parents scored significantly lower than controls on Cognitive empathy, Emotional empathy and Social skills latent factors as well as on the performance-based tasks. Individuals with autism also scored significantly lower than controls on all four latent factor means. There was a significant difference between parents and individuals with ASC on Cognitive empathy, Emotional empathy and Social skills factors. However, these two groups scored similarly on the performance tasks (Mean difference, -0.30, *P* >0.05). Please note that the current paper focused on latent factor means. Group differences on means of the different tasks under study have been reported elsewhere [6,20,27,37-39,59].

**Table 2:** Mean differences on factors scores in the multigroup CFA model

	**Cognitive empathy**	**Emotional empathy**	**Social skills**	**Cognitive tests**
	**Mean (95% CI)**	**Mean (95% CI)**	**Mean (95% CI)**	**Mean (95% CI)**
Control group (n = 232)	-0.04 (-0.16 to 0.08)	-0.06 (-0.16 to 0.04)	-0.03 (-0.15 to 0.10)	0.01 (-0.11 to 0.13)
Parent group (n = 439)	-0.36^a^ (-0.51 to -0.22)	-0.27^b^ (-0.44 to -0.10)	-0.38^a^ (-0.52 to -0.24)	-0.29^b^ (-0.55 to -0.04)
Autism group (n = 363)	-2.01^a^ (-2.25 to -1.78)	-1.21^a^ (-1.42 to -0.99)	-2.76^a^ (-3.05 to -2.46)	-0.59^a^ (-0.74 to -0.44)

^a^*P* <0.01 ^b^*P* <0.05 significantly different to controls.

The correlation between the performance-based test factor and the other empathy factors varied by sample group (see Table 3). In the control group, the performance-based tasks were not significantly correlated with any other empathy factor. However, in both the parent and ASC group these tasks were significantly correlated with Cognitive empathy, Emotional empathy and Social skills. These correlations were of similar magnitude for all factors. To verify that these different correlation patterns between the performance-based test factor and the other empathy factors in the groups could not be explained by differences in score distributions on the performance-based tasks, the distributions of the KDEF and Eyes tasks were inspected. Both tasks showed very similar distributions in the control and parent groups. The differences in the correlation patterns are therefore unlikely to be due to differences in the test score distributions.

**Table 3:** Correlation between the cognitive test factor and the other components of empathy

	**Cognitive empathy**	**Emotional empathy**	**Social skills**
	***r *** **(95% CI)**	***r *** **(95% CI)**	***r *** **(95% CI)**
Control group (n = 232)	0.15 (-0.10 to 0.39)	-0.04 (-0.23 to 0.17)	0.20 (-0.10 to 0.49)
Parent group (n = 439)	0.48^a^ (0.27 to 0.70)	0.49^a^ (0.25 to 0.74)	0.46^a^ (0.25 to 0.68)
Autism group (n = 363)	0.32^a^ (0.20 to 0.45)	0.32^a^ (0.18 to 0.45)	0.32^a^ (0.20 to 0.44)

^a^*P* <0.01.

## Discussion

Factor analyses in data from a large sample of individuals with ASC, parents and controls, using both questionnaire and performance-based measures of empathy, suggested a four-factor latent structure of empathy encompassing Cognitive empathy, Emotional empathy, Social skills and a Performance-based measurement factor. This structure was consistent across individuals deemed to have a high (individuals on the autism spectrum), medium (parents) or low (controls) genetic vulnerability for autism, indicating that the overall latent structure of empathy is consistent across both clinical and general population samples. However, there were some differences in the factor loadings and factor correlations across the three groups.

The latent structure identified in this study is consistent with previous research in that it identifies both a cognitive and emotional component of empathy [3,4,13,22]. In addition, the analyses also identified a separate Social skills factor. Items measured by the Social skills factor of the EQ assess specific empathising skills within a social situation. For example, ‘I find it hard to know what to do in a social situation’ and ‘I often find it difficult to judge if something is rude or polite’. Future research utilising other measures is needed to further assess the theoretical implications of this Social skills factor, which is shown to be separate from cognitive and emotional empathy.

It was expected that the performance-based emotion recognition tasks would be related to the Cognitive empathy factor. However, factor loadings of the Eyes and KDEF on the Cognitive empathy factor were low, with a model including a separate performance-based task component providing a better fit. Interestingly, the relationship between the Performance-based test factor and the other empathy factors was different across the three groups under study. In the control group, the performance tasks were not significantly correlated with any of the questionnaire-based empathy factors. Within parents and individuals with autism, the performance measures were related in almost equal magnitude to all three components, rather than solely to cognitive empathy. The finding that these performance tasks do not directly and exclusively assess cognitive empathy is new. Previous research has operated on the assumption that these tasks are performance-based measures of cognitive empathy. The findings of the current study indicate that rather than being a direct measure of cognitive empathy, scores on performance-based tasks like the Eyes and the KDEF have a bearing on empathy more widely. Our results suggest that completion of either of these tasks requires engagement of more than just cognitive empathy abilities. Rather, impairment on these performance-based tasks is indicative of a broader impairment across all facets of empathy. This has important implications for future research involving the implementation of such tasks.

Individuals with autism showed greater impairment (as indexed by lower mean latent factor scores) across the Cognitive and Emotional empathy, Social skills and Performance-based empathy factors compared with controls. Similarly, the ASC group displayed greater impairment than parents across all factor means, with the exception of the Performance-based factor. This fits with the notion of autism as a disorder of empathy [7-9]. In contrast with some previous research [3,22-24], there was no evidence that individuals with autism exhibited intact or heightened emotional empathy.

Parents also showed mild impairment across all four factors compared to controls. However, with the exception of the Performance-based factor, impairment was not as strong as observed in the ASC group, placing their difficulties somewhere in between the clinical and the control group. This is consistent with previous accounts indicating that first-degree relatives show some difficulties on tasks of empathy [20] compared with controls. Moreover, it fits with the notion that characteristics related to autism are distributed as quantitative traits rather than discrete entities [37,60] and are likely to be influenced at least in part by common genetic variation [28].

### Limitations

As mentioned previously, a number of fathers had missing data on the Eyes and KDEF tasks (n = 104). To assess whether these missing data had any effect on the results, all analyses were run both by imputing data for these individuals as well as excluding the missing cases. As there were no substantive changes within any of the models, it is highly unlikely that these missing data were a confounding factor.

The parent group also consisted of a larger proportion of mothers (n = 298) than fathers (n = 141). To ensure these differences would not bias the analyses, any sex effects on the means were regressed out prior to conducting the factor analyses. Moreover, the evaluation of sex differences in the latent factor structure indicated that it was similar across both sexes. Future studies including very large sample sizes would be of interest, as these could explore any possible sex difference in the latent factor structure in more detail than the current sample size permitted.

Lastly, the control group included in this study had completed a somewhat higher level of education than the parent and ASC groups. We can therefore not exclude the possibility that differences in educational level may explain some of the differences in factor structure of empathy observed between controls and the parent and ASC groups.

## Conclusions

The current study assessed the latent structure of empathy across individuals with a low, medium and high genetic vulnerability to autism. Results highlighted that empathy shows evidence of multidimensionality, in which four factors can be distinguished irrespective of genetic vulnerability, including three components of empathy and a performance-based factor. Unexpectedly, performance-based measures of empathy were related in almost equal magnitude to Cognitive empathy, Emotional empathy and Social skills, rather than solely to Cognitive empathy. This has implications for the nature of impairment indicated by performance on such tasks, suggesting that these effects are much wider than impairments in cognitive empathy alone. Individuals with autism displayed impairment on all four components of empathy, confirming the notion that autism is characterised by difficulties with multiple facets of empathy. Parents showed intermediate impairments of empathy, providing evidence for the BAP and highlighting the importance to assess characteristics of autism on quantitative scales.

## Abbreviations

AIC: Akaike information criterion; AQ: Autism Spectrum Quotient; AQ_soc: Social interaction factor of the Autism Spectrum Quotient; ASC: Autism spectrum conditions; BAP: Broader autism phenotype; BIC: Bayesian information criterion; CFA: Confirmatory factor analysis; CFI: Comparative fit index; E-S theory: Empathising-Systemising Theory; EQ: Empathy Quotient; Eyes: Reading the Mind in the Eyes test revised; KDEF: Karolinska Directed Emotional Faces; RMSEA: Root mean square error of approximation; SSABIC: Sample size adjusted Bayesian information criterion; ToM: Theory of Mind; TLI: Tucker Lewis index.

## Competing interests

The authors declare that they have no competing interests.

## Authors’ contributions

RG, RH and AB designed the study. Data reported on in this study were collected by CA and SB-C. RG undertook all statistical analyses and drafted the manuscript. All other authors contributed to subsequent drafts and the final submitted manuscript. All authors read and approved the final version of the manuscript.
